# Alteration of Metabolic Profile and Potential Biomarkers in the Plasma of Alzheimer’s Disease

**DOI:** 10.14336/AD.2020.0217

**Published:** 2020-12-01

**Authors:** Yaping Shao, Yang Ouyang, Tianbai Li, Xinyao Liu, Xiaojiao Xu, Song Li, Guowang Xu, Weidong Le

**Affiliations:** ^1^Center for Clinical Research on Neurological Diseases, The First Affiliated Hospital, Dalian Medical University, Dalian, China.; ^2^Liaoning Provincial Key Laboratory for Research on the Pathogenic Mechanisms of Neurological Diseases, The First Affiliated Hospital, Dalian Medical University, Dalian, China.; ^3^CAS Key Laboratory of Separation Science for Analytical Chemistry, Dalian Institute of Chemical Physics, Chinese Academy of Sciences, Dalian, China.; ^4^University of Chinese Academy of Sciences, Beijing, China

**Keywords:** Alzheimer’s disease, metabolomics, biomarker, metabolic pathway alteration, plasma

## Abstract

The expending of elderly population worldwide has resulted in a dramatic rise in the incidence of chronic diseases such as Alzheimer’s disease (AD). Inadequate understanding of the mechanisms underlying AD has hampered the development of efficient tools for definitive diagnosis and curative interventions. Previous studies have attempted to discover reliable biomarkers of AD, but these biomarkers can only be measured through invasive (neuropathological markers in cerebrospinal fluid) or expensive (positron emission tomography scanning or magnetic resonance imaging) techniques. Metabolomics is a high-throughput technology that can detect and catalog large numbers of small metabolites and may be a useful tool for characterization of AD and identification of biomarkers. In this study, we used ultra-performance liquid chromatography-mass spectrometry based untargeted metabolomics to measure the concentrations of plasma metabolites in a cohort of subjects with AD (n=44) and cognitively normal controls (Ctrl, n=94). The AD group showed marked reductions in levels of polyunsaturated fatty acids, acyl-carnitines, degradation products of tryptophan, and elevated levels of bile acids compared to the Ctrl group. We then validated the results using an independent cohort that included subjects with AD (n=30), mild cognitive impairment (MCI, n=13), healthy controls (n=43), and non-AD neurological disease controls (NDC, n=31). We identified five metabolites comprising cholic acid, chenodeoxycholic acid, allocholic acid, indolelactic acid, and tryptophan that were able to distinguish patients with AD from both Ctrl and NDC with satisfactory sensitivity and specificity. The concentrations of these metabolites were significantly correlated with disease severity. Our results also suggested that altered bile acid profiles in AD and MCI might indicate early risk for the development of AD. These findings may allow for development of new approaches for diagnosis of AD and may provide novel insights into AD pathogenesis.

Alzheimer’s disease (AD) is a progressive neuro-degenerative disorder that accounts for 60-80% of all dementia cases. Inadequate understanding of the etiology of AD and confounding factors that contribute to its genotypic and phenotypic heterogeneities may explain the lack of development of curative therapies [1, 2]. Genetic causes account for only 1-5% of all AD cases, and >95% of cases are sporadic [3]. Clinical diagnosis of AD is primarily reached based on medical history, patient symptoms, neuroimaging, and neuropsychological evaluation, and typically occurs too late for effective disease modification [4]. Although several methods have been developed for diagnosis of AD, including quantification of amyloid-β (Aβ) and tau protein levels in cerebrospinal fluid, measurement of Aβ using positron-emission tomography imaging, and analysis of atrophy using magnetic resonance imaging, these procedures are either invasive (lumbar puncture) or expensive (imaging) [5-7]. Therefore, identification of peripheral blood-derived biomarkers capable of identifying AD during early stages is an urgent priority.

Numerous studies have identified blood biomarkers of AD, the majority of which were derived from known disease markers such as Aβ and tau, which represents narrow hypothesis-driven biomarker discovery [8, 9]. Although many studies have proposed several biomarker candidates such as inflammation or oxidative stress-related proteins, cytokines, chemokines, growth factors, and lipids, there is still a clear need for a broader study of biomarker that could facilitate better understanding of the disease mechanisms [6, 10, 11]. Identification of novel biological characteristics of AD could contribute to the discovery of novel circulating biomarkers associated with risk for development of AD, and identification of therapeutic targets.

Metabolomics is a rapidly expanding field in systems biology used to explore the molecular basis of biological aspects of diseases [12]. Increasing numbers of studies have shown that dysregulation of various metabolic pathways, such as cholesterol metabolism and energy metabolism, may contribute to onset of AD pathology and AD-associated cognitive impairment [13]. Characterization of the role of metabolism in AD etiology using large-scale metabolomics could contribute to comprehensive understanding of AD pathology and discovery of mechanisms triggering symptom occurrence [13]. A previous study showed that altered plasma arginine metabolism preceded behavioral and brain metabolic profile changes in APP/PS1 transgenic mice [14]. Several studies have reported alterations in phospholipids, amine metabolites, and amino acids in the blood of AD patients [7, 12, 15, 16]. However, most of these studies were based on relatively small sample size, and the results were not validated, which makes comparative assessments of these studies difficult [17]. Moreover, traditional metabolomics-based studies of AD have mainly relied on comparisons between patients with AD/mild cognitive impairment (MCI) and healthy controls, and ignored potential interference from other neurological diseases, which has resulted in difficulty in determination of specificity of potential biomarkers.

In this study, we used a robust ultra-performance liquid chromatography-mass spectrometry (UPLC-MS)-based untargeted metabolomics approach to profile the plasma metabolome in AD. By comparing the metabolic differences between patients with AD and cognitively normal controls (Ctrl) using biostatistical and bioinformatics approaches, we highlighted specific biochemical pathways that were perturbed in AD. To further validate our findings and identify promising biomarker candidates, we repeated our analysis in an independent cohort that included patients with AD, MCI, Ctrl, and non-AD neurological disease controls (NDC). We developed a predictive model capable of distinguishing AD from Ctrl and NDC subjects and provided a novel perspective for early risk assessment of AD (Fig. 1).

**Figure 1.**
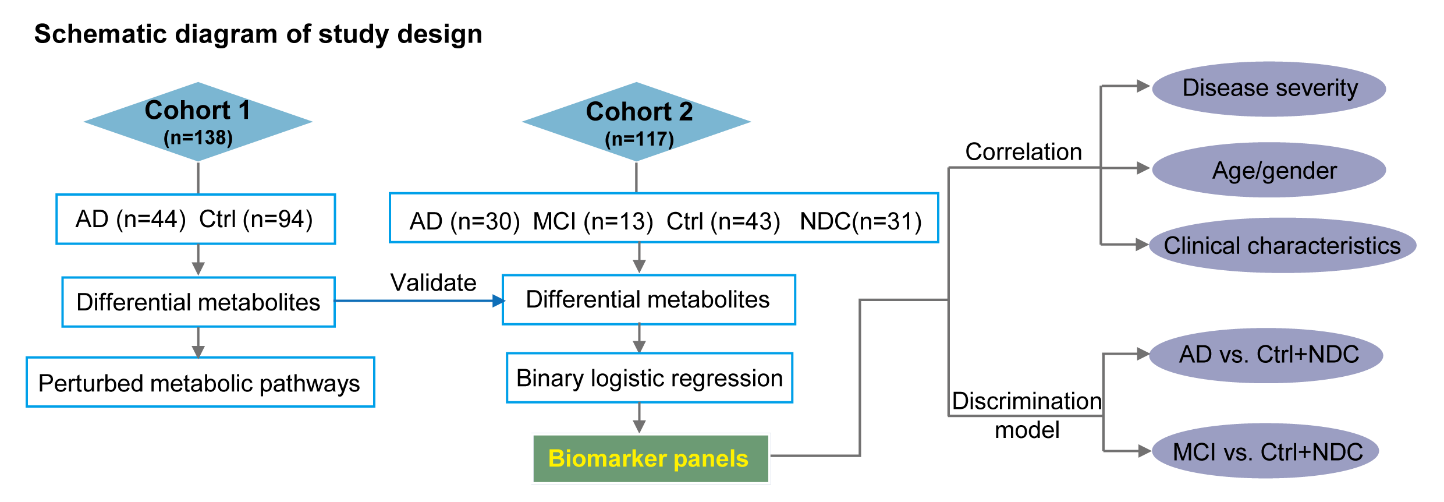
Experimental design. An overview workflow of the metabolomics analysis in Alzheimer's disease. A total of 255 plasma samples were collected and subjected to untargeted metabolomics analysis. Amony them, 138 and 117 plasma samples were included in discovery phase and validation phase, respectively.

## MATERIALS AND METHODS

### Study cohorts and patients

Fasting plasma samples were collected at the First Affiliated Hospital of Dalian Medical University. Two hundred fifty-five plasma samples were collected, and all subjects or their legally authorized caregivers provided written informed consent. In the discovery phase, we collected 138 plasma samples, of which 44 were from patients with AD and 94 were from Ctrl subjects. These samples were selected solely based on sample availability. In the validation phase, 117 plasma samples were collected from patients with AD (n = 30), Ctrl subjects (n = 43), NDC subjects (n = 31; 10 seizure, 10 migraine, 9 peripheral neuropathy, and 2 essential tremor), and patients with MCI (n = 13). Patients with AD and patients with MCI were subjected to standard cognitive screening including medical history assessment, physical, cognitive (Mini-mental State Examination (MMSE) scale and Montreal Cognitive Assessment (MoCA) scale) and neurologic examinations, routine blood and urine tests, and magnetic resonance imaging. AD diagnoses were made based on the National Institute of Neurological and Communicative Disorders and Stroke-Alzheimer’s Disease and Related Disorders Association criteria [18], and MCI was diagnosed using the criteria of Petersen [19, 20]. Patients with non-AD neurological diseases were diagnosed by experienced physicians from the Department of Neurology. This study was approved by the Ethics Committee of the First Affiliated Hospital of Dalian Medical University. Patient characteristics are presented in Table 1.

**Table 1:** Clinical information of the subjects in the study.

	AD (n=74)	Ctrl (n=137)	NDC (n=31)	MCI (n=13)	*p*
Cohort 1					
Number of individuals	44	94	-	-	-
Age, mean ± SE	72.0 ± 1.3	68.6 ± 0.8	-	-	0.0156 ^a^
Gender (m/f)	20/24	51/43	-	-	0.3350 ^b^
Duration of disease (year) ^c^, mean ± SE	4.0 ± 0.5	-	-	-	-
Disease progression^d^ early/middle/late stage	17/10/3	-	-	-	-
MMSE, mean ± SE ^e^	15.2 ± 1.0	-	-	-	-
MoCA, mean ± SE ^f^	10.9 ± 0.8	-	-	-	-
Cohort 2					
Number of individuals	30	43	31	13	-
Age, mean ± SE	71.6 ± 1.6	65.5 ± 1.2	62.2 ± 1.8	67.9 ± 2.0	0.0011
Gender (m/f)	10/20	25/18	17/14	8/5	0.1459
Duration of disease (year), mean ± SE	4.5 ± 0.5	-	-	3.9 ± 0.8	0.5852 ^g^
Disease progression early/middle/late stage	14/9/4	-	-	-	-
MMSE, mean ± SE	15.4 ± 1.5	-	-	25.1 ± 1.0	<0.0001
MoCA, mean ± SE	10.8 ± 1.5			20.0 ± 1.5	0.0003

a Student t-test and one-way ANOVA were applied to calculate the statistical significance of age between AD and Ctrl in cohort 1 as well as AD, Ctrl, NDC and MCI in cohort 2, respectively. Notably, there is no significant difference among Ctrl, NDC and MCI with a *p* value of 0.1134 calculated by one-way ANOVA.

b Chi-square test was used to calculate the significant difference of gender composition between groups.

c Three AD patients in cohort 1 and one AD patient, one MCI patient in cohort 2 were lack of information of disease duration.

d Information of disease progression were not available for fourteen AD patients in cohort 1 and three AD patients in cohort 2.

e,f Twelve AD patients in cohort 1 and ten AD patients, four MCI patients in cohort 2 were lack of MMSE and MoCA data.

g Student t-test was applied to calculate the statistical significance of duration of disease, MMSE and MoCA between AD and MCI. AD, Alzheimer's disease; Ctrl, cognitively normal controls; MCI, mild cognitive impairment; NDC, non-AD neurological diseases controls; MMSE, Mini-mental State Examination; MoCA, Montreal Cognitive Assessment.

### Preparation of plasma samples

Blood samples were collected in ethylene-diaminetetraacetic acid-containing vacutainer tubes by direct venipuncture, then immediately centrifugated at 3,000 rpm for 5 min. Plasma was transferred to sterile tubes, stored at -80°C, and thawed on ice prior to analysis. Plasma metabolites were extracted as previously described [21, 22]. One hundred-thirty microliters of plasma was deproteinated using 520 μL of methanol that contained 14 internal standards (carnitine C2:0-d3, carnitine C10:0-d3, carnitine C16:0-d3, lysophosphatidyl-choline (LPC) 12:0, leucine-d3, phenylalanine-d5, tryptophan-d5, cholic acid-d4, chenodeoxycholic acid-d4, choine-d4, palmitic acid-d3, stearic acid-d3, succinic acid-d4, and tridecanoic acid), then vortexed for 1 min. After centrifugation for 15 min (14,000 g, 4?), the supernatant was divided into two aliquots, transferred to fresh tubes, then dried in a vacuum centrifuge. Prior to LC-MS analysis, the dried powder was reconstituted in 65 μL of 25% (v/v) aqueous methanol.

A biological quality control (QC) sample was made by pooling 10-20 μL of each plasma sample. QC samples were prepared in parallel with study samples and analyzed after 10 sample injections to monitor the robustness of the large-scale analysis.

**Figure 2.**
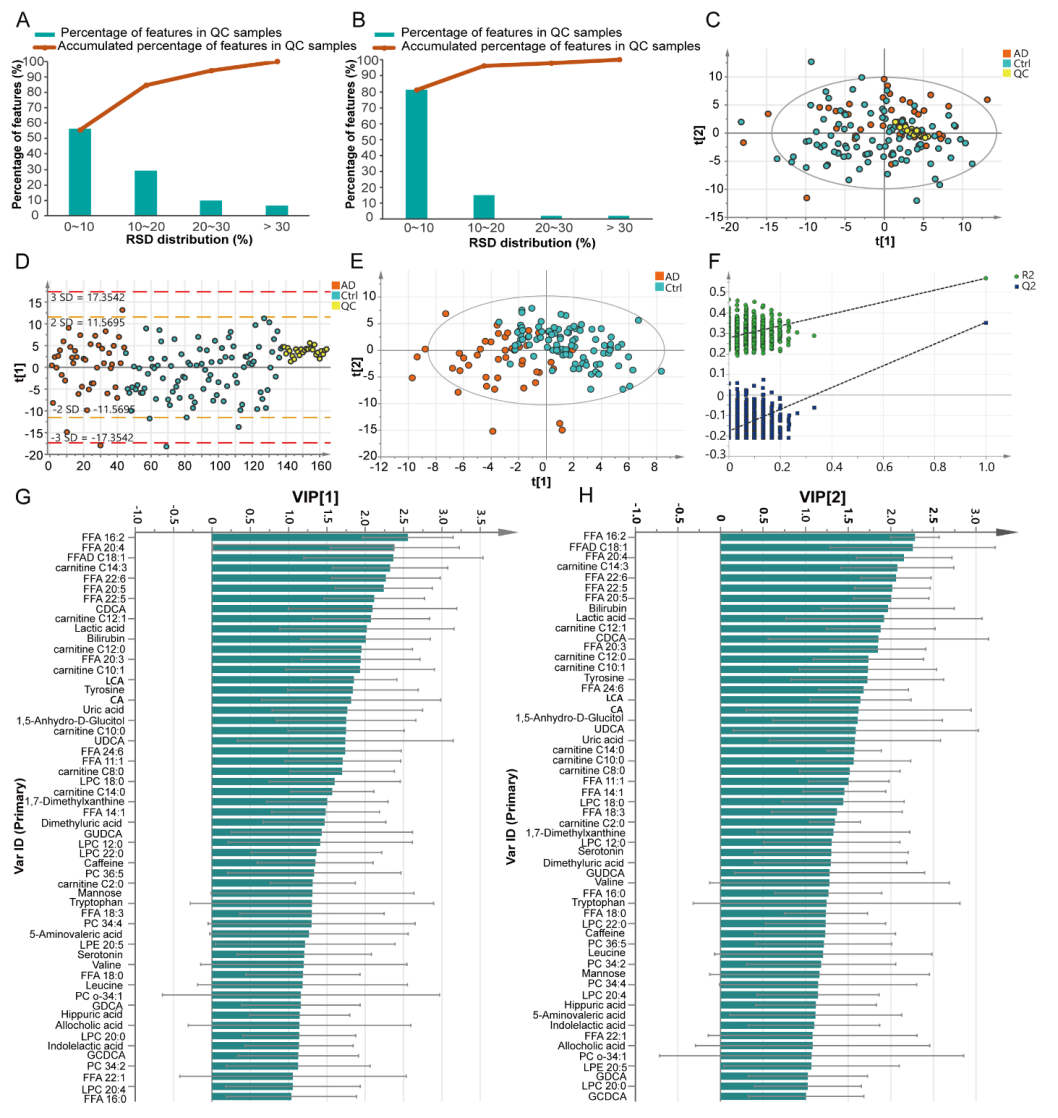
Quality control of the analytical method and multivariate statistical analysis. (A) RSD distribution of all the detected features in the QC samples. (B) RSD distribution of the identified metabolites in the QC samples. (C) PCA score plot. QC samples clustered tightly on the plot, indicating good quality control of the analytical method. (D) Standard deviation of the samples. Most of the samples were located within ±2SD. (E) Score plot of the PLS-DA model. Model parameters: R2Y=0.575, Q2=0.427. (F) Permutation test of the PLS-DA model. 999 permutations resulted in intercepts of R2 = 0.266, Q2 = -0.175, indicating an acceptable model without overfitting. (G) Metabolites with VIP [1] score > 1 in PLS-DA model. (H) Metabolites with VIP [2] score > 1 in PLS-DA model.

### Metabolomic profiling

Untargeted metabolomic profiling was performed using UPLC (Waters Corporation, Manchester, UK) coupled to a tripleTOF™5600 plus (Applied Biosystems, Foster City, CA) mass spectrometry system. For electrospray ionization positive (ESI+) mode detection, an ACQUITY UPLC BEH C8 1.7 μm column was used. The mobile phases were 0.1% formic acid in water and acetonitrile. For ESI negative (ESI-) mode detection, an ACQUITY UPLC HSS T3 1.8 μm column was used. The mobile phases were 6.5 mM NH_4_HCO_3_ in water and 95% methanol (v/v) with 6.5 mM NH_4_HCO_3_. The gradient elution profile and mass spectrometry parameters were published in our previous studies [21, 23].

### Metabolite recognition and data preprocessing

Metabolite identification was performed using an in-house database that includes about 2,000 metabolites [24]. QC samples were used to generate qualitative information including retention time, accurate mass (*m/z*) and MS2 spectrum by performing automatic MS2 scans in information-dependent acquisition mode. Retention time calibration, precursor ion and auto-MS2 information matching were performed using OSI/SMMS software. Peak detection and alignment were conducted using Marker View software (version 1.2.1.1, Applied Biosystems). After refining peaks using the 80% rule [25], the original peak area of each retained peak was calibrated by internal standards prior to statistical analysis. For each peak, the internal standard was selected to obtain the minimum relative standard deviation (RSD) for the peak in the QC samples. For metabolites detected and identified in the discovery and validation phases, the original peak area was calibrated using the same internal standards.

### Statistical analysis

Multivariate analyses were performed using SIMCA 13.0 (Umetrics AB, Umea, Sweden). An unsupervised model of principal component analysis (PCA) was used to assess the stability of the analytical process and to visualize global metabolome differences among the groups. To maximize class discrimination necessary for discovery of potential metabolic biomarkers between the AD and Ctrl groups, a supervised model of partial least squares discriminant analysis (PLS-DA) was applied. The variable importance in the projection (VIP) value was used to estimate the discriminatory power of each variable for separation of groups in the PLS-DA model, and variables with VIP values > 1 were considered important. Furthermore, we performed a permutation test to ensure that the PLS-DA model was not overfitting.

Univariate analyses utilized Student’s t-test (*p* < 0.05). A heat map was constructed using Multi Experiment Viewer (Version 4.7.4) software to visualize the concentrations of metabolites in different groups. To determine the metabolic pathway changes relevant to the AD disease process, we performed pathway enrichment analysis using the online software MetaboAnalyst (www.metaboanalyst.ca). Binary logistic regression analysis was applied to establish a potential metabolite panel, and a summary receiver operating characteristic (ROC) curve was generated to evaluate the predictive performance of the model. Spearman correlation was used to determine associations between categorical variables (disease severity/gender) and levels of metabolites. Pearson correlation was used to determine associations among continuous variables (age/clinical characteristics) using SPSS 18.0 software (SPSS, Inc., USA).

## RESULTS

To define differential metabolites and perturbed metabolic pathways that differentiated AD from neurologically normal controls, we performed an untargeted metabolomics approach using plasma samples from 44 AD patients and 94 Ctrl individuals. Totally, 2,412 ion signatures in ESI+ mode and 2,672 ion signatures in ESI- mode were detected in both AD and Ctrl groups. Using our in-house database, 208 metabolites were identified. The RSD of QC samples was used as an indicator of analytical reproducibility. The results showed that 93.8% of all detected peaks had RSD values < 30% (Fig. 2A), and 98.1% of the identified peaks had RSD values < 30% (Fig. 2B), which indicated that the analysis was repeatable and robust. The 208 identified ion signatures were further analyzed using an unsupervised, multivariate classification technique. QC samples clustered tightly on the PCA score plot (Fig. 2C), and most samples (98.2%, 161/164) were within the 95% confidence interval (CI) in the direction of the first principal component (Fig. 2D), which further confirmed the reliability of this study.

### Discovery of differential metabolites and dysregulation of metabolic pathways in AD

**Figure 3.**
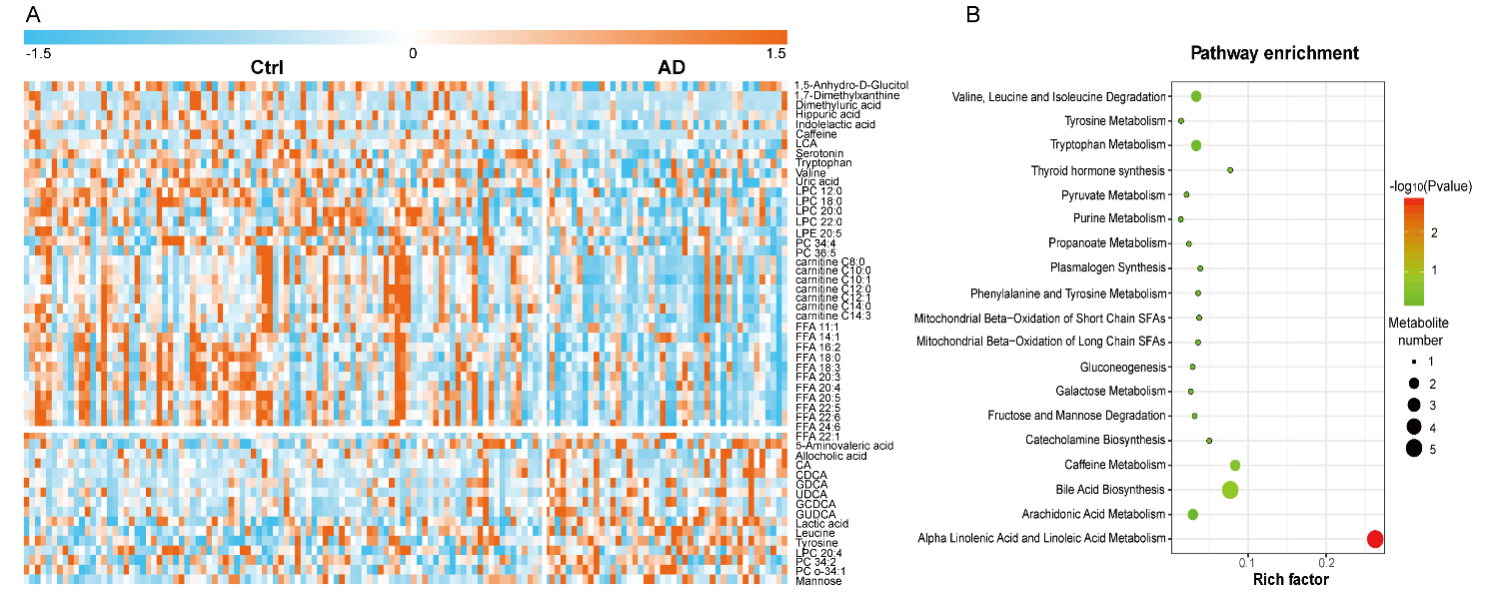
Differential metabolites and perturbed metabolic pathways in AD. (A) Heat map of the significantly changed metabolites in AD compared with Ctrl. (B) Pathway enrichment analysis based on differential metabolites.

To visualize differences within and between groups, and to determine important metabolites that could be used to distinguish AD and Ctrl, a PLS-DA model with unit variance scaling was established. The plasma metabolomes of AD and Ctrl were significantly different (Fig. 2E). The model was confirmed to not be overfitted following 999 permutation tests (Fig. 2F). Among the 208 identified metabolites, 56 metabolites were found to be important to the separation of AD and Ctrl with both VIP [1] and VIP [2] values > 1 (Fig. 2G-2H). These 56 metabolites were subjected to univariate analysis (Student’s t-test), and 52 of them were found to be statistically significant (*p* < 0.05). The levels of these 52 metabolites were visualized in a heat map (Fig. 3A). Because of the age imbalance between the Ctrl and AD group, we further investigated correlations between levels of metabolites and age. Of the 52 differential metabolites, only phosphatidylcholine (PC) O-34:1 showed a significant correlation with age in the Ctrl group (*p* = 0.0013, r = 0.327). Therefore, the differences in metabolic levels between AD and Ctrl were not owing to age difference between the two groups. Most of the differential metabolites, including acylcarnitines, fatty acids (FAs), several LPCs (12:0, 18:0, 20:0, 22:0) and PCs (C34:4 and C36:5), amino acids (valine, tryptophan), serotonin, and several organic acids, were reduced in AD. In contrast, bile acids including allocholic acid, cholic acid (CA), chenodeoxycholic acid (CDCA), glycol-chenodeoxycholic acid (GDCA), ursodeoxycholic acid (UDCA), glycochenodeoxycholic acid (GCDCA), and glycoursodeoxycholic acid (GUDCA), and lactic acid, leucine, and tyrosine were elevated in AD. Furthermore, pathway enrichment analysis showed that alpha linolenic acid and linoleic acid metabolism, bile acid biosynthesis, caffeine metabolism, thyroid hormone synthesis, and catecholamine biosynthesis were the most significantly perturbed pathways in AD (Fig. 3B).

### Differential metabolites validation and metabolite panel establishment

**Figure 4.**
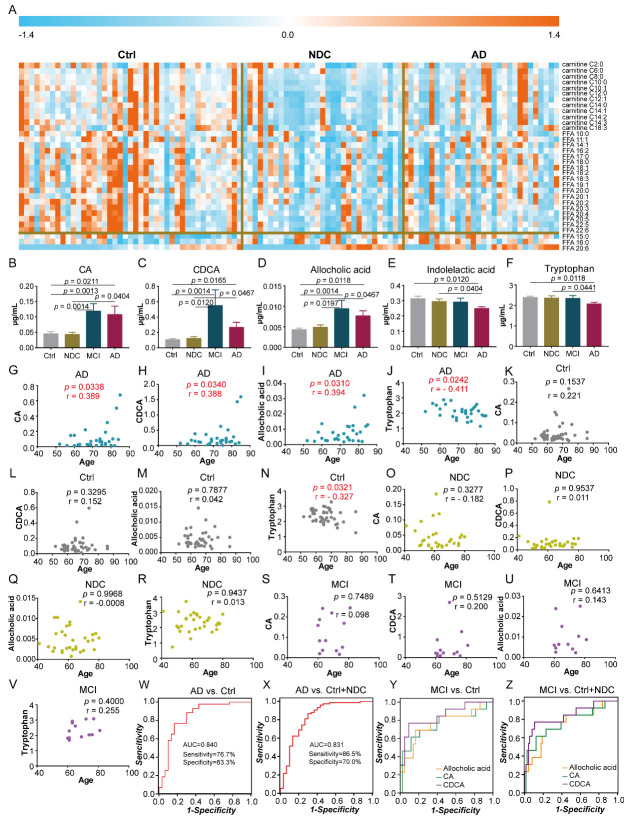
Metabolite panel and ROC analysis. (A) Heat map of FAs and acyl-carnitines in Ctrl, NDC and AD. (B) ~ (F) Bar graphs of the 5 metabolites in different groups. The *p* values were adjusted for multiple testing using Benjamini-Hochberg method. (G) ~ (V) Correlation analysis of the five metabolites with age in AD, Ctrl, NDC and MCI. (W) ~ (X) ROC analysis of the metabolite panels to discriminate AD from Ctrl/(Ctrl +NDC). (Y) ~ (Z) ROC analysis of bile acids to discriminate MCI from Ctrl/(Ctrl +NDC).

To further validate the specificity of these differential metabolites in AD, we collected and analyzed an independent cohort of plasma samples from 30 AD, 43 Ctrl, 31 NDC, and 13 MCI cases. In the validation phase, we observed markedly lower levels of FAs and acyl-carnitines in AD compared to those in the Ctrl group, which was consistent with previous results. However, we found that patients in the NDC group showed a greater decrease in the levels of FAs and acyl-carnitines, which indicated that changes in these metabolites were not specific (Fig. 4A). Following comparisons between AD and Ctrl, as well as AD and NDC, levels of five metabolites were found to be specifically altered in AD. The levels of CA, CDCA, and allocholic acid were increased, while the levels of indolelactic acid and tryptophan were decreased in AD compared with those in the Ctrl and NDC groups (Fig. 4B-4F).

ROC curves of classification models based on the 5 specific metabolites were plotted to distinguish AD from control groups. The area-under-the-curve (AUC) values for each metabolite were 0.721, 0.700, 0.691, 0.643, and 0.632, respectively. The discriminating power was improved by combining these 5 metabolites into a metabolite panel using binary logistic regression analysis, as evidenced by an AUC value of 0.822. Because of the imbalance in age distribution between AD and the other three groups (Ctrl, MCI and NDC), we further analyzed the correlations between the selected metabolites and age in each group. We found that none of the five metabolites showed significant correlations with age in NDC or MCI, whereas the levels of CA, CDCA, allocholic acid, and tryptophan were significantly associated with age in AD (Fig. 4G-4V). Therefore, we also included age as a covariate for development of a binary logistic regression model. The corresponding ROC curve showed an AUC value of 0.840 with 76.7% sensitivity and 83.3% specificity (Fig. 4W). Moreover, this metabolite panel also showed satisfactory diagnostic performance for distinguishing AD from both Ctrl and NDC with AUC, sensitivity, and specificity of 0.831, 86.5%, and 70.0%, respectively (Fig. 4X). Notably, we found that changes in bile acids were more significant in patients MCI (Fig. 4B-4D). Using CDCA alone to differentiate MCI from Ctrl resulted in an AUC value of 0.850 with a 95% CI of 0.721-0.979 (Fig. 4Y). Furthermore, CDCA alone produced a satisfactory AUC value of 0.839 for differentiating MCI from both Ctrl and NDC groups with a 95% CI of 0.705-0.973 (Fig. 4Z). The discriminating power of each bile acid metabolite was presented in Table 2.

**Table 2:** Results for assessment of plasma metabolite panel in the discrimination of AD/MCI.

	Metabolite panel	AUC	95% CI	Sensitivity	Specificity	SE	Significance
Meta+age	AD vs. Ctrl	0.840	0.740 ~ 0.941	76.7%	83.3%	0.0512	8.57E-07
	AD vs. (Ctrl+NDC)	0.831	0.733 ~ 0.928	86.5%	70.0%	0.0496	1.39E-07
Allocholic acid	MCI vs. Ctrl	0.766	0.604 ~ 0.928	69.2%	83.7%	0.0827	3.95E-03
	MCI vs. (Ctrl+NDC)	0.745	0.591 ~ 0.900	69.2%	77.0%	0.0787	4.96E-03
CA	MCI vs. Ctrl	0.758	0.585 ~ 0.932	61.5%	88.4%	0.0887	5.04E-03
	MCI vs. (Ctrl+NDC)	0.770	0.612 ~ 0.928	61.5%	87.8%	0.0807	1.96E-03
CDCA	MCI vs. Ctrl	0.850	0.721 ~ 0.979	76.9%	90.7%	0.0659	1.48E-04
	MCI vs. (Ctrl+NDC)	0.839	0.705 ~ 0.973	76.9%	89.2%	0.0684	1.04E-04

CI, confidence interval; SE, standard error.

### Associations of metabolites with gender, disease severity, and clinical characteristics

**Figure 5.**
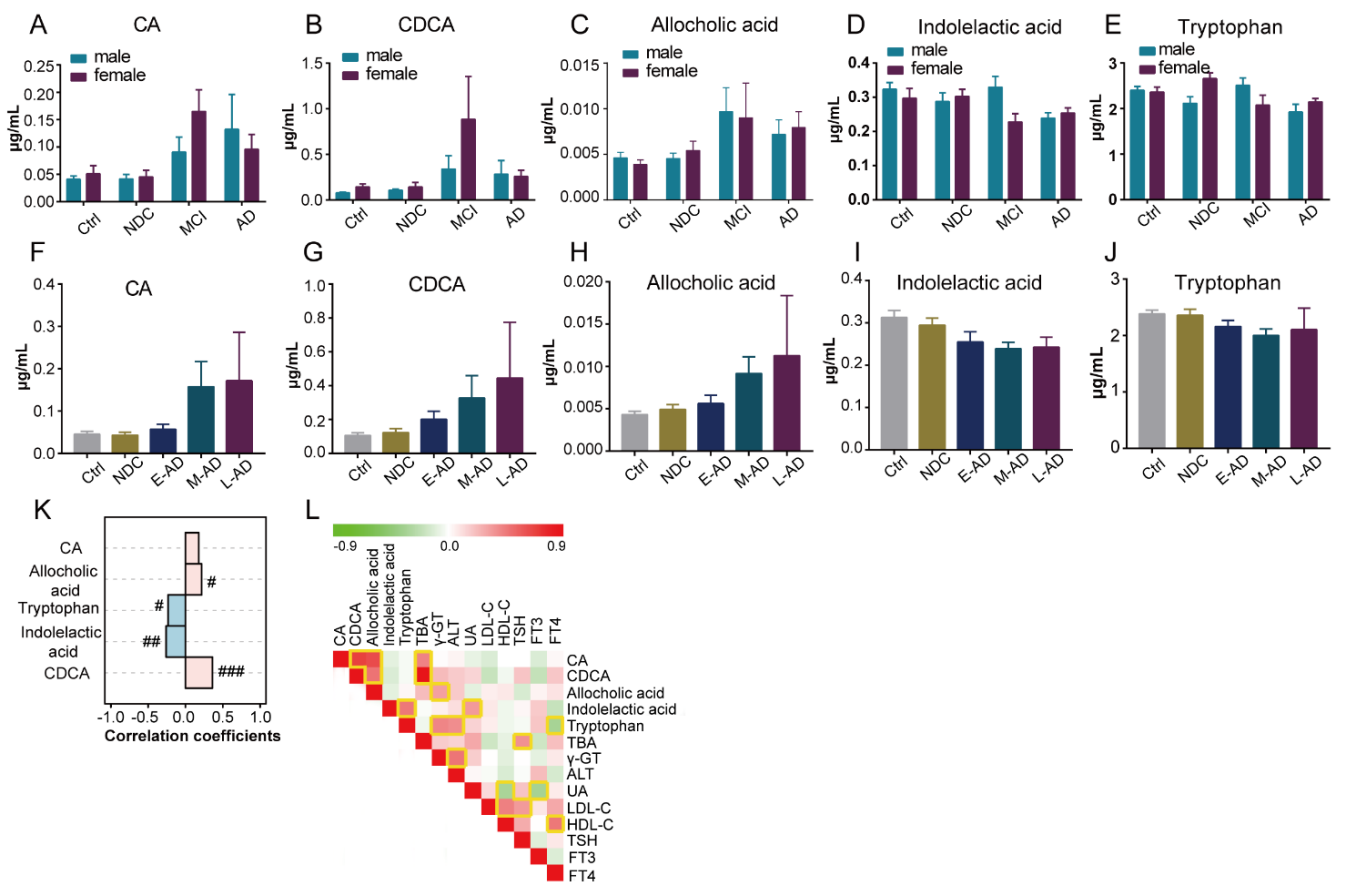
Correlation analysis of the metabolites panel with gender, disease severity and clinical characteristics. (A) ~ (E) Distribution of the levels of metabolite in different genders in Ctrl, NDC, MCI and AD. (F) ~ (K) Correlation analysis of the metabolite levels to disease progress. Correlation coefficients were based on Spearman correlation analysis. #, 0.01 < *p* < 0.05, ##, 0.001 < *p* <0.01, ###, 0.0001 < *p* < 0.001. (L) Correlations analysis of metabolite levels with clinical parameters, which was based on Pearson correlation analysis. Yellow square indicated the correlations between two variables were statistically significant.

No significant correlations were found between the five selected metabolites and gender (Fig. 5A-5E). Interestingly, the levels of CDCA and allocholic acid were positively correlated with disease severity of AD, and the levels of indolelactic acid and tryptophan were negatively correlated with disease severity (Fig. 5F-5K). Furthermore, correlation analyses of the levels of metabolites with clinical characteristics were performed to characterize the relationship between plasma metabolic profiles and the clinical phenotype of AD. We found that the levels of CA and CDCA were positively correlated with total bile acids (TBA), and the levels of allocholic acid were positively correlated with γ-glutamyltransferase (γ-GT). Furthermore, indolelactic acid was positively correlated with uric acid (UA), while tryptophan levels were positively correlated with γ-GT and alanine aminotransferase (ALT), and negatively correlated with free thyroxine 4 (FT4) (Fig. 5L). Associations between the selected metabolites and MMSE and MoCA scores were also investigated in AD patients. However, no significant correlations were observed (Supplementary Table 1).

## DISCUSSION

In this study, we employed untargeted metabolomics and biostatistical approaches to gain further insight into metabolic network failures and to identify potential metabolic biomarkers in AD. A total of 255 plasma samples (AD, n = 74; MCI, n = 13; Ctrl, n = 137; NDC, n = 31) were collected and analyzed. First, we compared the plasma metabolomes of AD patients with matched cognitively normal individuals. Our results suggested that the overall metabolic expression profile differed between the two groups. Following VIP value filtering and multivariate and univariate analyses, 52 metabolites were found to be differentially altered in AD. Pathway enrichment analysis showed that alpha linolenic acid, linoleic acid, and arachidonic acid metabolism, mitochondrial beta-oxidation of saturated FAs, bile acid biosynthesis, and amino acid metabolism were the most relevant pathways associated with AD.

### Metabolic pathway dysregulations in AD

We observed marked reductions in the levels of FAs (except for C22:1). In particular, polyunsaturated fatty acids (PUFAs) levels were reduced in AD compared with those in Ctrl, which agreed with previous studies [26]. PUFAs are neuroprotective, and decreased levels of PUFAs may contribute to the cognitive decline associated with AD [27]. A recent study showed that unsaturated FA (UFA) metabolism was perturbed in the brains of AD patients. This study showed significant associations between UFA levels and neuritic plaques, neurofibrillary tangle burden, and cognitive performance [13]. Docosahexaenoic acid (DHA, C22:6) is the PUFA most associated with AD. Our results showed a reduction in DHA biosynthesis in AD, which corroborated the results from previous studies that reduced levels of DHA may be resulted from defective FA metabolism in the liver [27, 28]. Dietary supplementation with DHA has been shown to improve cognitive performance in several animal models of AD and in AD patients [29, 30]. PUFAs also play important roles in AD pathology. *In vivo* studies suggested that linoleic acid (C18:2), arachidonic acid (C20:4), and oleic acid (C18:1) induced polymerization of tau and Aβ and contributed to AD pathological progress [13, 31].

Additionally, we also noted that levels of nearly all acyl-carnitines were lower in AD than those in the Ctrl group, but only medium- and long-chain acyl-carnitines were significantly different between the two groups. A previous study indicated that serum acetyl-carnitine and other acyl-carnitine levels declined across the continuum from healthy individuals to subjects with subjective memory impairment and MCI, and patients with AD, which agreed with our findings [2]. Deficits in medium- and long-chain acyl-carnitines indicated disruption of FA transport into the mitochondria for beta-oxidation, and impaired energy metabolism [2]. These results also can be evidenced by the findings from proteomic and transcriptomic studies, which showed decreased carnitine shuttle activity and FA beta-oxidation in AD patients [32, 33]. Significant reductions in the levels of acyl carnitines were observed in the hippocampus and cerebral cortex of APP/PS1 transgenic mice [34]. Deficits in carnitine shuttling and beta-oxidation might be associated with mitochondrial dysfunction that contributed to the pathogenesis of AD.

In contrast to the decreased levels of FAs and acyl-carnitines, we observed increased levels of bile acids in AD, with the exception of lithocholic acid (LCA). The levels of CA, CDCA, and allocholic acid were increased with disease severity. Elevated levels of bile acids in the circulation usually result from increased bile acid synthesis and liver dysfunction [35], which was consistent with our findings of significant correlations between CA, CDCA and TBA and between allocholic acid and γ-GT. Recently, a larger multi-center study from the Alzheimer’s Disease Neuroimaging Initiative cohort used targeted metabolomic profiling, and reported strong associations between bile acid profiles and Aβ, tau, and neurodegeneration biomarkers, which suggested that the gut-liver-brain axis may play a role in AD pathogenesis [1, 36]. Primary bile acids are initially synthesized from cholesterol in the liver, and are converted to secondary bile acids by intestinal anaerobic bacteria [1]. In addition to potential effects from diet and cholesterol metabolism, conditions such as dysbiosis and antibiotic treatment might cause variations in bile acids levels [37]. Our study further supported a strong association between AD and abnormal bile acid metabolism. Further investigation of the underlying contributions of bile acids to the onset and progression of AD may result in a novel hypothesis of AD pathogenesis.

Perturbations in amino acid metabolism were also observed in our study. Reduced levels of tryptophan, serotonin, and indolelactic acid suggested that tryptophan degradation was enhanced through the kynurenine pathway in AD. Tryptophan is a precursor of several bioactive compounds including neurotransmitters (e.g., serotonin), and its metabolism plays an important role in regulating neuronal activity and the inflammatory response [38]. Furthermore, lower plasma tryptophan levels were associated with decreased olfactory function in elderly populations [38]. We observed a continuous decrease in levels of tryptophan and indolelactic acid with the progression of AD. In addition, tryptophan showed an age-related decrease in both AD and Ctrl groups, and significant correlations with γ-GT, ALT, and FT4 in our study, which suggested that tryptophan may be a clinical marker of disease risk in the elderly. Recently, an *in vitro* study showed that tryptophan, serotonin, melatonin, and other indole compounds, protected against Aβ peptide aggregation and cytotoxicity [39]. Investigation of the possible mechanism underlying the neuroprotective effects of indole compounds may result in identification of novel potential therapeutic targets for AD.

### Metabolite panels for AD discrimination

To screen stable and specific biomarkers for AD discrimination, we repeated our analysis in an independent cohort that included AD, Ctrl, MCI, and NDC groups to further validate previous findings. Inclusion of the NDC group as a disease control was intended to provide an additional control for evaluation of biomarker specificity. According to our results, reduced levels of FAs and acyl-carnitines in the AD and NDC groups limited their use as diagnostic biomarkers for AD. We identified a metabolite panel comprised of five metabolites including CA, CDCA, allocholic acid, indolelactic acid, and tryptophan that may allow for discrimination of subjects with AD from cognitively normal and non-AD neurological disease controls.

In addition to serving as potential diagnostic biomarkers, the five AD-specific markers identified in this study showed significant correlations with disease progression, which suggested that these markers or the pathways from which they derive may be potential targets for treatment of AD. In addition, significant differences in levels of bile acids, particularly CDCA, in MCI suggested that disruption of bile acid profiles may serve as a potential indicator of AD risk. These results agreed with those of previous studies that demonstrated strong associations between bile acid profiles and cognitive impairment in AD [36, 40].

In summary, combination of metabolomics with statistical and bioinformatic analyses resulted in identification of overall metabolic network failures in AD. Our results indicated that dysregulation of PUFA metabolism, mitochondrial β-oxidation, bile acid synthesis, and tryptophan metabolism were pivotal events in AD progression. Further investigation of the specific roles of these metabolic changes in the pathogenesis of AD might result in development of novel therapeutic strategies. However, further studies are needed to validate our findings. Although a validation phase was included in our study, the samples size is still insufficient, and future validation studies should include multi-center clinical trials with larger sample sizes. Furthermore, metabolomics-based longitudinal studies of patients with AD in the pre-symptomatic phase will be crucial to the identification of novel biomarkers for early diagnosis and early intervention.

## Supplementary Materials

The Supplemenantry data can be found online at: www.aginganddisease.org/EN/10.14336/AD.2020.0217.

## References

[1] MahmoudianDehkordi S, Arnold M, Nho K, Ahmad S, Jia W, Xie G, et al (2019). Altered bile acid profile associates with cognitive impairment in Alzheimer's disease-An emerging role for gut microbiome. Alzheimers Dement, 15:76-92. 30337151 10.1016/j.jalz.2018.07.217 PMC6487485

[2] Cristofano A, Sapere N, La Marca G, Angiolillo A, Vitale M, Corbi G, et al (2016). Serum Levels of Acyl-Carnitines along the Continuum from Normal to Alzheimer's Dementia. PLoS One, 11:e0155694. 27196316 10.1371/journal.pone.0155694 PMC4873244

[3] Reitz C, Mayeux R (2014). Alzheimer disease: epidemiology, diagnostic criteria, risk factors and biomarkers. Biochem Pharmacol, 88:640-651. 24398425 10.1016/j.bcp.2013.12.024 PMC3992261

[4] Xu XH, Huang Y, Wang G, Chen SD (2012). Metabolomics: a novel approach to identify potential diagnostic biomarkers and pathogenesis in Alzheimer's disease. Neurosci Bull, 28:641-648. 23054640 10.1007/s12264-012-1272-0 PMC5561924

[5] Bayer AJ (2018). The role of biomarkers and imaging in the clinical diagnosis of dementia. Age Ageing, 47:641-643. 29432519 10.1093/ageing/afy004

[6] Morgan AR, Touchard S, Leckey C, O'Hagan C, Nevado-Holgado AJ, Barkhof F, et al (2019). Inflammatory biomarkers in Alzheimer's disease plasma. Alzheimers Dement, 15:776-787. 31047856 10.1016/j.jalz.2019.03.007 PMC6565806

[7] Lin CN, Huang CC, Huang KL, Lin KJ, Yen TC, Kuo HC (2019). A metabolomic approach to identifying biomarkers in blood of Alzheimer's disease. Ann Clin Transl Neurol, 6:537-545. 30911577 10.1002/acn3.726 PMC6414491

[8] Startin CM, Ashton NJ, Hamburg S, Hithersay R, Wiseman FK, Mok KY, et al (2019). Plasma biomarkers for amyloid, tau, and cytokines in Down syndrome and sporadic Alzheimer's disease. Alzheimers Res Ther, 11:26. 30902060 10.1186/s13195-019-0477-0 PMC6429702

[9] Nabers A, Hafermann H, Wiltfang J, Gerwert K (2019). Abeta and tau structure-based biomarkers for a blood- and CSF-based two-step recruitment strategy to identify patients with dementia due to Alzheimer's disease. Alzheimers Dement (Amst), 11:257-263. 30911600 10.1016/j.dadm.2019.01.008 PMC6416642

[10] Patra K, Soosaipillai A, Sando SB, Lauridsen C, Berge G, Møller I, et al (2018). Assessment of kallikrein 6 as a cross-sectional and longitudinal biomarker for Alzheimer’s disease. Alzheimers Res Ther, 10:9. 29378650 10.1186/s13195-018-0336-4 PMC5789599

[11] Balietti M, Giuli C, Conti F (2018). Peripheral Blood Brain-Derived Neurotrophic Factor as a Biomarker of Alzheimer's Disease: Are There Methodological Biases? Mol Neurobiol, 55:6661-6672. 29330839 10.1007/s12035-017-0866-y PMC6061178

[12] Tynkkynen J, Chouraki V, van der Lee SJ, Hernesniemi J, Yang Q, Li S, et al (2018). Association of branched-chain amino acids and other circulating metabolites with risk of incident dementia and Alzheimer's disease: A prospective study in eight cohorts. Alzheimers Dement, 14:723-733. 29519576 10.1016/j.jalz.2018.01.003 PMC6082422

[13] Snowden SG, Ebshiana AA, Hye A, An Y, Pletnikova O, O'Brien R, et al (2017). Association between fatty acid metabolism in the brain and Alzheimer disease neuropathology and cognitive performance: A nontargeted metabolomic study. PLoS Med, 14:e1002266. 28323825 10.1371/journal.pmed.1002266 PMC5360226

[14] Bergin DH, Jing Y, Mockett BG, Zhang H, Abraham WC, Liu P (2018). Altered plasma arginine metabolome precedes behavioural and brain arginine metabolomic profile changes in the APPswe/PS1DeltaE9 mouse model of Alzheimer's disease. Transl Psychiatry, 8:108. 29802260 10.1038/s41398-018-0149-z PMC5970225

[15] Mapstone M, Cheema AK, Fiandaca MS, Zhong X, Mhyre TR, MacArthur LH, et al (2014). Plasma phospholipids identify antecedent memory impairment in older adults. Nat Med, 20:415-418. 24608097 10.1038/nm.3466 PMC5360460

[16] Chouraki V, Preis SR, Yang Q, Beiser A, Li S, Larson MG, et al (2017). Association of amine biomarkers with incident dementia and Alzheimer's disease in the Framingham Study. Alzheimers Dement, 13:1327-1336. 28602601 10.1016/j.jalz.2017.04.009 PMC5722716

[17] Casanova R, Varma S, Simpson B, Kim M, An Y, Saldana S, et al (2016). Blood metabolite markers of preclinical Alzheimer's disease in two longitudinally followed cohorts of older individuals. Alzheimers Dement, 12:815-822. 26806385 10.1016/j.jalz.2015.12.008 PMC4947451

[18] Mckhann G, Drachman D, Folstein MF, Katzman R, Price D, Stadlan E (1984). Clinical diagnosis of Alzheimer disease: report of NINCDS-ADRDA Work Group under the auspices of Department of Health and Human Service Task Force on Alzheimer's Disease. Neurology, 34:939-944. 6610841 10.1212/wnl.34.7.939

[19] Petersen RC, Smith GE, Waring SC, Ivnik RJ, Tangalos EG, Kokmen E (1999). Mild Cognitive Impairment: Clinical Characterization and Outcome. Arch Neurol, 56:303-308. 10190820 10.1001/archneur.56.3.303

[20] Petersen RC (2004). Mild cognitive impairment as a diagnostic entity. J Intern Med, 256:183-194. 15324362 10.1111/j.1365-2796.2004.01388.x

[21] Huang Y, Chen G, Liu X, Shao Y, Gao P, Xin C, et al (2014). Serum metabolomics study and eicosanoid analysis of childhood atopic dermatitis based on liquid chromatography-mass spectrometry. J Proteome Res, 13:5715-5723. 25316199 10.1021/pr5007069

[22] Luo P, Yin P, Hua R, Tan Y, Li Z, Qiu G, et al (2018). A Large-scale, multicenter serum metabolite biomarker identification study for the early detection of hepatocellular carcinoma. Hepatology, 67:662-675. 28960374 10.1002/hep.29561 PMC6680350

[23] Ren S, Shao Y, Zhao X, Hong CS, Wang F, Lu X, et al (2016). Integration of Metabolomics and Transcriptomics Reveals Major Metabolic Pathways and Potential Biomarker Involved in Prostate Cancer. Mol Cell Proteomics, 15:154-163. 26545398 10.1074/mcp.M115.052381 PMC4762514

[24] Zhao X, Zeng Z, Chen A, Lu X, Zhao C, Hu C, et al (2018). Comprehensive Strategy to Construct In-House Database for Accurate and Batch Identification of Small Molecular Metabolites. Anal Chem, 90:7635-7643. 29807420 10.1021/acs.analchem.8b01482

[25] Smilde AK, van der Werf MJ, Bijlsma S, van der Werff-van der Vat BJC, Jellema RH (2005). Fusion of mass spectrometry-based metabolomics data. Anal Chem, 77:6729-6736. 16223263 10.1021/ac051080y

[26] Cunnane SC, Schneider JA, Tangney C, Tremblay-Mercier J, Fortier M, Bennett DA, et al (2012). Plasma and brain fatty acid profiles in mild cognitive impairment and Alzheimer's disease. J Alzheimers Dis, 29:691-697. 22466064 10.3233/JAD-2012-110629 PMC3409580

[27] Astarita G, Jung KM, Berchtold NC, Nguyen VQ, Gillen DL, Head E, et al (2010). Deficient liver biosynthesis of docosahexaenoic acid correlates with cognitive impairment in Alzheimer's disease. PLoS One, 5:e12538. 20838618 10.1371/journal.pone.0012538 PMC2935886

[28] Thomas MH, Paris C, Magnien M, Colin J, Pelleieux S, Coste F, et al (2017). Dietary arachidonic acid increases deleterious effects of amyloid-beta oligomers on learning abilities and expression of AMPA receptors: putative role of the ACSL4-cPLA2 balance. Alzheimers Res Ther, 9:69. 28851448 10.1186/s13195-017-0295-1 PMC5576249

[29] Swanson D, Block R, Mousa SA (2012). Omega-3 fatty acids EPA and DHA: health benefits throughout life. Adv Nutr, 3:1-7. 22332096 10.3945/an.111.000893 PMC3262608

[30] Quinn JF, Raman R, Thomas RG, Yurko-Mauro K, Nelson EB, Van Dyck C, et al (2010). Docosahexaenoic acid supplementation and cognitive decline in Alzheimer disease: a randomized trial. JAMA, 304:1903-1911. 21045096 10.1001/jama.2010.1510 PMC3259852

[31] Amtul Z, Uhrig M, Wang L, Rozmahel RF, Beyreuther K (2012). Detrimental effects of arachidonic acid and its metabolites in cellular and mouse models of Alzheimer's disease: structural insight. Neurobiol Aging, 33:831.e821-831.e831. 10.1016/j.neurobiolaging.2011.07.014 21920632

[32] Stempler S, Yizhak K, Ruppin E (2014). Integrating transcriptomics with metabolic modeling predicts biomarkers and drug targets for Alzheimer's disease. PLoS One, 9:e105383. 25127241 10.1371/journal.pone.0105383 PMC4134302

[33] Dey KK, Wang H, Niu M, Bai B, Wang X, Li Y, et al (2019). Deep undepleted human serum proteome profiling toward biomarker discovery for Alzheimer's disease. Clin Proteomics, 16:16. 31019427 10.1186/s12014-019-9237-1 PMC6472024

[34] Raúl GD, Tamara GB, Javier V, José Luis GA (2015). Metabolomic screening of regional brain alterations in the APP/PS1 transgenic model of Alzheimer's disease by direct infusion mass spectrometry. J Pharm Biomed Anal, 102:425-435. 25459942 10.1016/j.jpba.2014.10.009

[35] Xie G, Wang X, Huang F, Zhao A, Chen W, Yan J, et al (2016). Dysregulated hepatic bile acids collaboratively promote liver carcinogenesis. Int J Cancer, 139:1764-1775. 27273788 10.1002/ijc.30219 PMC5493524

[36] Nho K, Kueider-Paisley A, MahmoudianDehkordi S, Arnold M, Risacher SL, Louie G, et al (2019). Altered bile acid profile in mild cognitive impairment and Alzheimer's disease: Relationship to neuroimaging and CSF biomarkers. Alzheimers Dement, 15:232-244. 30337152 10.1016/j.jalz.2018.08.012 PMC6454538

[37] Pan X, Elliott CT, McGuinness B, Passmore P, Kehoe PG, Hölscher C, et al (2017). Metabolomic Profiling of Bile Acids in Clinical and Experimental Samples of Alzheimer’s Disease. Metabolites, 7:28. 10.3390/metabo7020028 PMC5487999 28629125

[38] Adachi Y, Shimodaira Y, Nakamura H, Imaizumi A, Mori M, Kageyama Y, et al (2017). Low plasma tryptophan is associated with olfactory function in healthy elderly community dwellers in Japan. BMC Geriatr, 17:239. 29037152 10.1186/s12877-017-0639-5 PMC5644149

[39] Hornedo-Ortega R, Da Costa G, Cerezo AB, Troncoso AM, Richard T, Garcia-Parrilla MC (2018). In Vitro Effects of Serotonin, Melatonin, and Other Related Indole Compounds on Amyloid-β Kinetics and Neuroprotection. Mol Nutr Food Res, 62:1700383. 10.1002/mnfr.201700383 29131485

[40] Marksteiner J, Blasko I, Kemmler G, Koal T, Humpel C (2018). Bile acid quantification of 20 plasma metabolites identifies lithocholic acid as a putative biomarker in Alzheimer's disease. Metabolomics, 14:1. 29249916 10.1007/s11306-017-1297-5 PMC5725507

