# Supplementary material for: Alteration of Metabolic Profile and Potential Biomarkers in the Plasma of Alzheimer’s Disease
Source: Aging Dis. 2020 Dec 1;11(6):1459–70. doi: 10.14336/AD.2020.0217 (PMC7673846; doi:10.14336/AD.2020.0217)
Supplement: Supplementary file 1 — The Supplemenantry data can be found online at: www.aginganddisease.org/EN/10.14336/AD.2020.0217. [file AD-11-6-1459-s.pdf]

SUPPLEMENTARY DATA

Alteration of Metabolic Profile and Potential Biomarkers in the Plasma of Alzheimer's Disease

Yaping Shao[1, 2], Yang Ouyang[3, 4], Tianbai Li[1, 2], Xinyao Liu[1, 2], Xiaojiao Xu[1, 2], Song Li[1, 2], Guowang Xu[3*], Weidong Le[1, 2*]

© 2020. Shao Y et al. Published online at http://www.aginganddisease.org/EN/10.14336/AD.2020.0217

SUPPLEMENTARY DATA

Supplementary Table 1. Statistical results of correlations between the five selected metabolites and MMSE/MoCA.

		CA	CDCA	Allocholic acid	Indolelactic acid	Tryptophan
MMSE	r	- 0.172	- 0.189	- 0.062	- 0.135	0.166
	p value	0.470	0.425	0.794	0.569	0.485
MoCA	r	- 0.077	- 0.172	0.045	- 0.172	0.148
	p value	0.748	0.713	0.849	0.468	0.533

Based on Pearson correlation analysis. r: correlation coefficient; MMSE, Mini-mental State Examination; MoCA, Montreal Cognitive Assessment.

© 2020. Shao Y et al. Published online at http://www.aginganddisease.org/EN/10.14336/AD.2020.0217
